# Effects of Siliceous Natural Nanomaterials Applied in Combination with Foliar Fertilizers on Physiology, Yield and Fruit Quality of the Apricot and Peach Trees

**DOI:** 10.3390/plants10112395

**Published:** 2021-11-06

**Authors:** Cristina Moale, Marius Ghiurea, Carmen Eugenia Sîrbu, Raluca Somoghi, Traian Mihai Cioroianu, Victor Alexandru Faraon, Carmen Lupu, Bogdan Trică, Diana Constantinescu-Aruxandei, Florin Oancea

**Affiliations:** 1 Research Station for Fruit Growing Constanța, Str. Pepinierei nr. 25, 907300 Valul lui Traian, Romania; moalecristina@yahoo.com; 2 Department of Bioresources, National Institute for Research & Development in Chemistry and Petrochemistry—ICECHIM, Splaiul Independenței nr. 202, Sector 6, 060021 Bucharest, Romania; marius.ghiurea@icechim.ro (M.G.); raluca.somoghi@icechim.ro (R.S.); victor.faraon@icechim.ro (V.A.F.); carmen.lupu@icechim.ro (C.L.); bogdan.trica@icechim.ro (B.T.); diana.constantinescu@icechim.ro (D.C.-A.); 3 Department of Physico-Chemical Analysis, National Research and Development Institute for Soil Science, Agro-Chemistry and Environment—ICPA, Mărăşti Blvd. nr. 61, Sector 1, 011464 Bucharest, Romania; carmen.sirbu@icpa.ro (C.E.S.); traian.cioroianu@icpa.ro (T.M.C.); 4 Faculty of Biotechnologies, University of Agronomic Sciences and Veterinary Medicine of Bucharest, Mărăşti Blvd. nr. 59, Sector 1, 011464 Bucharest, Romania

**Keywords:** diatomaceous earth, natural zeolites, foliar fertilizer, leaf physiology, photosynthesis, yield, fruit quality, polyphenol accumulation

## Abstract

Siliceous natural nanomaterials (SNNMs), i.e., diatomaceous earth and natural zeolites, have a nanoporous structure with large active surfaces that adsorb cations or polarized molecules. Such nanoporous feature determines the effects related to SNNM utilization as low-risk plant protectants and soil improvers. This work used SNNMs from Romanian quarries as carriers for foliar fertilizers applied to stone-fruit trees, apricot and peach. We determined the effects of SNNMs on the physiology, yield and fruit quality of the treated stone-fruit trees. SNNM application determined impacts specific to the formation of particle films on leaves: reduced leaf temperature (up to 4.5 °C) and enhanced water use efficiency (up to 30%). Foliar fertilizers’ effects on yield are amplified by their application with SNNMs. Yield is increased up to 8.1% by the utilization of SNNMs with foliar fertilizers, compared to applying foliar fertilizer alone. Diatomaceous earth and natural zeolites promote the accumulation of polyphenols in apricot and peach fruits. The combined application of SNNMs and foliar fertilizer enhance the performance of peach and apricot trees.

## 1. Introduction

Siliceous natural nanomaterials (SNNMs), i.e., diatomaceous earth/kieselguhr and natural zeolites, are used in agriculture as biorational—low-risk plant protectants, plant biostimulants/plant strengtheners or soil amenders/soil improvers [1]. Their main applications are related to their characteristic features: large volumes of free space and active surfaces, with cation exchange capacity and/or reversible sorption of polarized molecules, including water [2,3,4].

SNNMs’ characteristic features result from their structure that includes nanopores/nanocavities and nanochannels/nanotunnels. The nanoporous and nanotunneled structure of diatomaceous earth (DE) is a consequence of its (bio)genesis. DE is a sedimentary rock, resulting from the fossilized cell walls (shells) of diatoms, photosynthetic microorganisms included in the *Heterokonta* infrakingdom. Diatom cell shells (also called frustules) are 3D nanopatterned structures of amorphous biosilica, SiO_2_*xn*H_2_O. Biomolecules associated with diatom cell walls, such as cingulin and silaffin proteins or long-chain polyamines (LCPAs), precipitate amorphous biosilica [5]. The mineralization of these biomolecules’ microrings determines the nanopatterned structure of DE—nanopores/nanocavities with connecting nanotunnels/nanochannels [6].

Natural zeolites are inorganic polymers—and their inorganic polymer nature determines their nanoporous structure. Natural zeolites are crystalline hydrated polymeric tectoaluminosilicates [7]. The monomeric units are tetrahedral units—TO4, where T is aluminum (Al) or silicon (Si) [8]. Oxygen bonds covalently bind these tetrahedral units [9]. These -O- bonded tetrahedrons generate a honeycomb structure at the nano-level, with nanopores/nanocavities and nanochannels/nanotunnels. [2]. The capacity to release soluble silicon species was also proven for natural zeolites. A Si-rich mineral zeolite increased barley plant tolerance against cadmium stress [10].

Desiccant activity, resulting from the active surfaces with water sorption capacity and large volumes of pores and interconnected nanotunnels, is responsible for the activity against plant pathogens and pests from stored grains [3] or leaves [11]. Large volumes of free space and active surfaces, with cation exchange capacity and/or polarized molecules, are involved in the improving effects of SNNMs on soils or growing media [12,13]. Adding DE or natural zeolites [14] into the soil or growing media improves water holding capacity and cation exchange capacity (CEC). Nitrogen use efficiency is improved after applying SNNMs to soils or growing substrates due to fixation and slow release of nitrogen species [15]. SNNM CEC also is related to the utilization of macro-nutrients, e.g., potassium [16,17], oligo-nutrients, e.g., zinc [18,19], and micro-nutrients, e.g., manganese [19]. The interaction between silicate and phosphates is involved in the beneficial effects of natural zeolites applied to soil on crop yield and soil phosphate leaching [20,21].

Until now, SNNMs were not yet used as slow-release carriers for foliar-applied fertilizers. Foliar fertilization is considered a more targeted and sustainable approach to applying the mineral nutrients needed for crop yield and quality [22]. Foliar fertilization still needs optimization in terms of the mineral uptake of hydrophilic nutrients through the hydrophobic surface of the leaves [23]. Significant improvements in foliar fertilization were considered to result from nanoformulation of the foliar fertilizer [24] and foliar nutrient chelation [25]. SNNMs bind the nutrients to active surfaces located in nanostructures and, therefore, should improve foliar fertilization. Our main objective for this work was to determine the effects of SNNMs on foliar fertilization efficiency in two species of stone-fruit trees, apricot and peach. Such perennial fruit trees significantly benefit from applying foliar fertilizer [24]. Apricot, *Prunus armeniaca* L., was chosen because this crop is more prone to highly variable yield [26]—and the application of SNNMs in combination with foliar fertilizers should buffer such erratic yields. Peach, *P. persica* (L.) Batsch, was selected due to the significant influence of the nutrients on fruit quality attributes [27].

Another objective of this work was to investigate the effects of SNNMs with foliar fertilizers on the physiology of the treated fruit trees, apricot and peach. SNNMs form a particle film on the surface of the leaves. Such particle films determine beneficial effects on treated plants by decreasing leaves’ temperature and transpiration rate and enhancing photosynthesis and gas exchange [28]. The particle film effects result from a combination of physical and physiological mechanisms. Decrease in the leaf temperature reduces the harmful effects of heat on chloroplasts—inactivation of Rubisco (D-ribulose 1, 5-bisphosphate carboxylase–oxygenase, first enzyme of the Calvin cycle), chlorophyll breakdown, inactivation of PSII and impairment of protein translation [28]. SNNMs can also concentrate CO_2_ close to stomata [28]. DE and natural zeolites were demonstrated to act as reversible CO_2_ (chemo)sorbents [29,30]. Selective enrichment with CO_2_ of the space near stomata should enhance CO_2_ fixation, especially on C3 plants. However, such enhanced effect on the photosynthesis of SNNMs was demonstrated only in one publication for zeolite formulations [31]. DE application on Chinese cabbage reduced photosynthesis and inhibited secondary metabolism [32]. Application of another siliceous nanomaterial, kaolin, as a reflective anti-transpirant [33] and a photosynthesis and gas-exchange promotor [34] enhances photosynthesis in sweet orange [35], red grapefruit [36], olive [34] and grape [37]. Photosynthesis inhibition was noted after kaolin dusting on tomatoes, apples [38], hazelnut and walnut [39], olives [40] and grapevine [41]. Italian natural chabazite-rich zeolite was reported to have no influence on photosynthesis in treated leaves of olive [40] and grapevine [41]. After foliar application of Italian natural chabazite-rich zeolite, improved olive or raisin quality was reported for olive trees [40] and grapevine [41].

Therefore, the effects of foliar application of the selected SNNMs, natural zeolites and DE, on fruit tree leaves are still scarce and contradictory and need further investigation. Our overall goal of this work was to determine the effects of the siliceous natural nanomaterials applied in combination with foliar fertilizers on the physiology, performance and fruit quality of the apricot and peach trees.

## 2. Results

### 2.1. Characterization of the Used Siliceous Natural Nanomaterials

**Figure 1:**
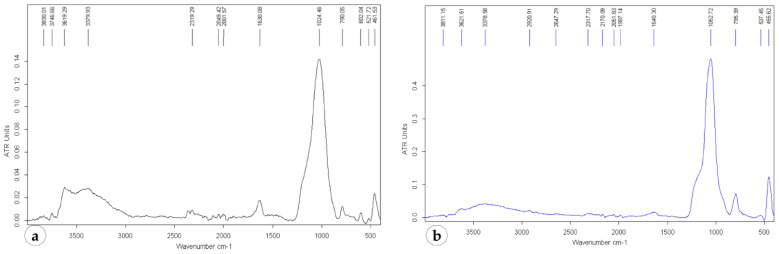
The ATR-FTIR spectra of the natural zeolites from Rupea quarry (**a**) and the diatomaceous earth from Pătârlagele quarry (**b**).

The ATR-FTIR spectra of the natural zeolites from the Rupea quarry and diatomaceous earth from the Pătârlagele quarry are presented in Figure 1.

FTIR analyses confirm the presence of functional group characteristics of zeolites and diatomaceous substances. The peak positions are approximately identical for all the samples investigated, confirming their siliceous structure. In natural zeolites from Rupea, the peaks from 462 cm^−1^ are attributed to the structure of the interconnected TO_4_ (T=Si or Al, four oxygen atoms surrounding a cation of silicon or aluminum) and to the bending vibration of the linked tetrahedra. Peaks from 521 cm^−1^ can be assigned to the stretching of the double ring of zeolites. Peaks from 790 cm^−1^ are attributed to the stretching bond vibration of the symmetrical (Si–O–Si) from linked tetrahedra. The peak at 1024 cm^−1^ is a consequence of the strong T–O stretching vibration band. The peak position suggests the presence of heulandite beside clinoptilolite in the analyzed natural zeolites [42]. The peaks around the values of 1630 and 3379 cm^−1^ are attributed to the bending of H–O–H bonds and the hydroxyl groups of the zeolite, respectively.

The peaks characteristic of diatomaceous earth samples are detected at 3378, 1640, 1053, 795 and 455 cm^−1^. The shoulder band from 3378 cm^−1^ is due to the stretching vibration of the hydroxyl groups of the physically adsorbed water molecules by silanol group Si-OH. This silanol group is responsible for the sorption–desorption process of water [43]. The IR carbonate band, OCO bending (in-plane deformation) mode [44], appears at 1640 cm^−1^. The in-plane vibration band of the asymmetric stretching of siloxane (–Si–O–Si–) in Pătârlagele diatomaceous earth [44,45] appears at the wavenumber of 1053 cm^−1^. Other bands at 795 and 455 cm^−1^ are also characteristic of silica; the first may be due to the stretching vibration of Al–O–Si but can also be attributed to the deformation of O–H or free silica and/or symmetrical stretching in Si-OH, while the second band is attributed to the bending vibrations of Si–O–Si.

**Figure 2:**
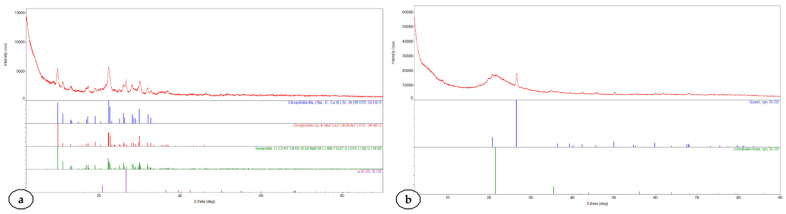
XRD diffractograms of crystalline fractions from Rupea natural zeolites (**a**) and Pătârlagele diatomaceous earth (**b**).

The XRD diffractograms of crystalline fractions from Rupea natural zeolites and Pătârlagele diatomaceous earth are presented in Figure 2.

**Figure 3:**
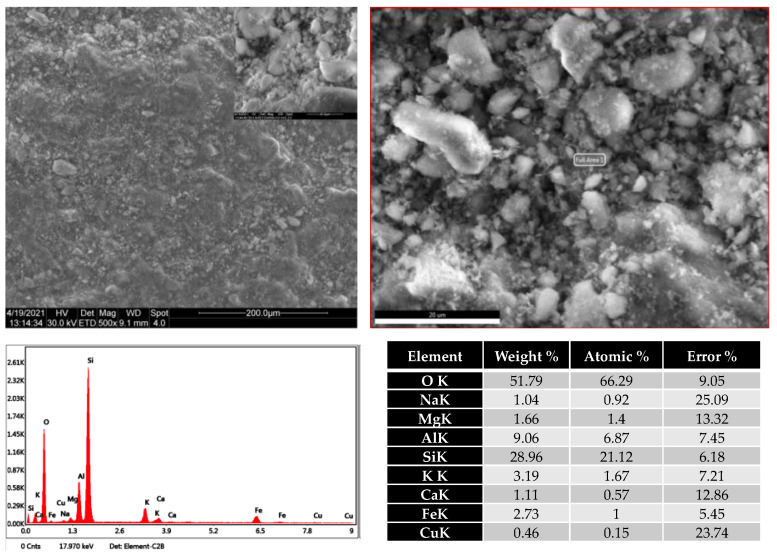
Scanning electron microscopy images of Rupea natural zeolites (upper left) and elemental analysis performed by EDX detector (down left). The upper right image represents the area analyzed with the EDX detector.

The XRD analysis confirmed the clinoptilolite as the significant crystalline fraction of natural zeolites from the Rupea quarry. This analysis confirmed the presence of heulandite beside clinoptilolite. The FTIR analysis also suggests this presence of heulandite. In the Pătârlagele DE, the crystalline fraction is quartz, with a low presence of cristobalite. The low occurrence of cristobalite indicates the safety of the diatomaceous earth from the Pătârlagele quarry—cristobalite being considered a significant threat to human health [46,47]. ESEM images of Rupea natural zeolite samples are shown in Figure 3, together with the elemental analysis performed by the EDX detector.

The surface relief of Rupea natural zeolite samples is highly heterogeneous. Enlargement of the image indicates the coexistence of crystals of various shapes and sizes, together with (poly)amorphous materials. The elemental analysis confirmed the presence of the aluminosilicates—oxygen, silicon and aluminum being the preponderant atoms.

**Figure 4:**
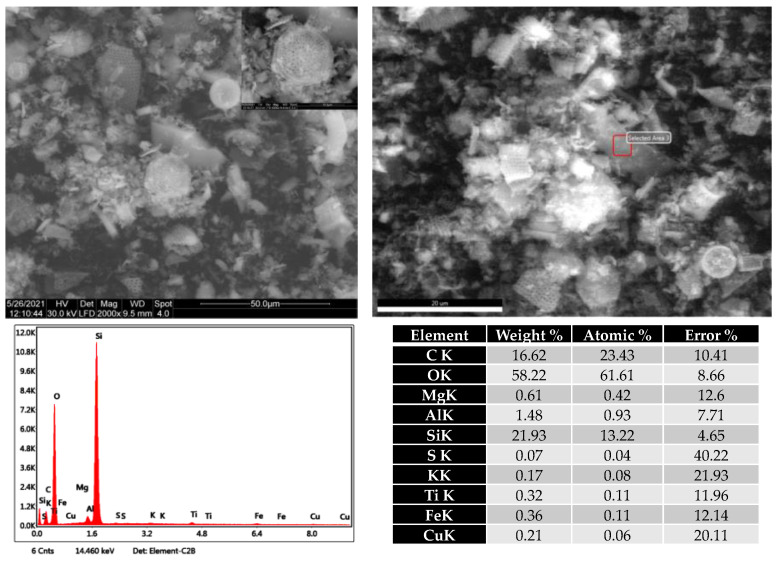
Scanning electron microscopy images of Pătârlagele diatomaceous earth (upper left) and elemental analysis performed by EDX detector (down left). The upper right image represents the area analyzed with the EDX detector.

Figure 4 presents the ESEM (environmental scanning electron microscopy) images of diatomaceous earth from the Pătârlagele quarry and the elemental analysis performed by EDX.

SEM images reveal the existence of pieces of diatom frustules as well as intact frustules. The frustules are deformed due to both compressional tectonics and soft-sediment deformation. The elemental composition indicates the predominance of SiO2 as a significant component of the analyzed DE but also the presence of the carbonate—that was also indicated by the FTIR analysis. The carbonate is (partially) eliminated during the activation treatment with hydrochloric acids.

**Figure 5:**
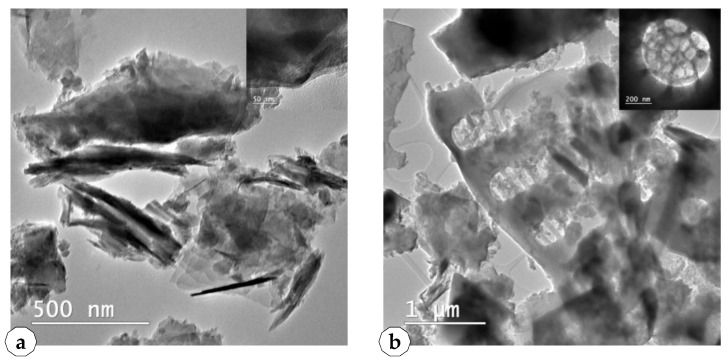
Transmission electron microscopy images of SNNMs, (**a**) Rupea natural zeolites, (**b**) Pătârlagele diatomaceous earth.

Transmission electron microscopy (TEM) images of SNNMs used in this study are presented in Figure 5.

TEM images confirm the existence of the crystals in the Rupea natural zeolites and the damaged frustules with high porosity in the Pătârlagele DE. The average estimated size of the crystal from Rupea natural zeolites is 50 nm. TEM demonstrated the porous structure of the diatom frustules, with large nanopores under 100 nm—Figure 5b, upper.

**Figure 6:**
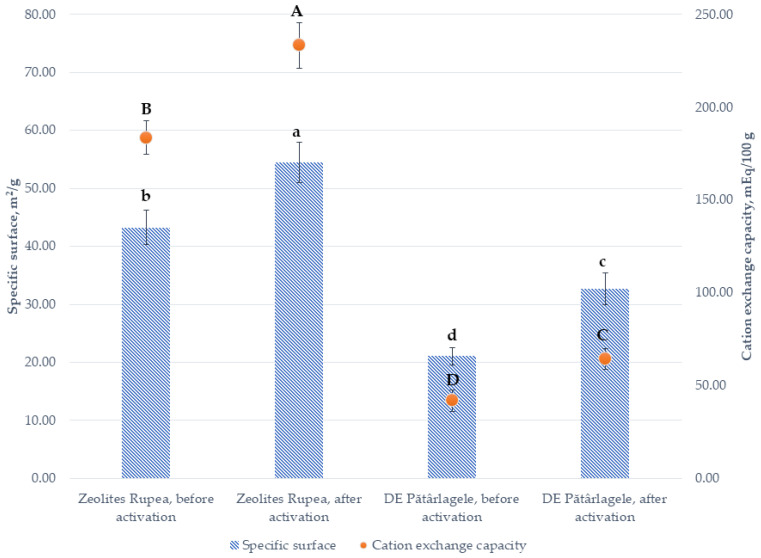
BET total surface areas and cation exchange capacity (CEC) of the two used SNNMs before and after activation treatment. The values presented represent means ± standard errors (*n* = 3 replicates). Data labeled with different letters within each parameter are significantly different at *p* < 0.05.

Figure 6 presents comparatively the BET (Brunauer–Emmett–Teller) total surface areas and cation exchange capacity (CEC) of the two used SNNMs before and after activation treatment.

The activation treatment increases BET total surface areas and CEC. The increase in the total BET surface area of natural zeolites from the Rupea quarry is 25.84%. In the case of diatomaceous earth from Pătârlagele, the enlargement of the specific surface is higher, of 55.34%. Such an increase is probably related to the carbonate dissolution after the acid treatment during the activation process.

After the activation process, the total cation exchange capacity of the natural zeolites from the Rupea quarry increased by 27.05%. In the case of DE from Pătârlagele, the total cation exchange capacity is higher, with 53.14% after the activation process.

The pore volume and average pore diameter were slightly modified after activation treatment—Table 1.

### 2.2. Analysis of the Foliar Fertilizer

The foliar fertilizer (called F1) and the foliar fertilizer with diatomaceous earth (NanoFert D) and natural zeolites (NanoFert Z) estimated and determined values are presented in Table 2, Table 3 and Table 4.

Molybdenum was not determined, and it was considered to be 0.012, according to the receipt for preparation of the foliar fertilizer. The differences between the estimated value (resulting from the proportion of the fertilizers introduced in the preparation process) and the determined value are within the limit established by the EU Regulation 2003/2003. The uncertainty values also follow the analytical standard used for fertilizers, presented in the EU Regulation 2003/2003 and the corresponding ISO EN Standards.

The data from Table 2, Table 3 and Table 4 demonstrated that the SNNM matrix, natural zeolites or diatomaceous earth did not significantly modify the foliar fertilizer composition. The determined composition is similar for the three analyzed foliar fertilizers, with or without the addition of SNNMs. In addition, the uncertainty value of the chosen analytical method is not significantly influenced by the SNNM matrix.

### 2.3. Effects of the SNNM Application on Physiological Characteristics of the Apricot and Peach Trees

**Figure 7:**
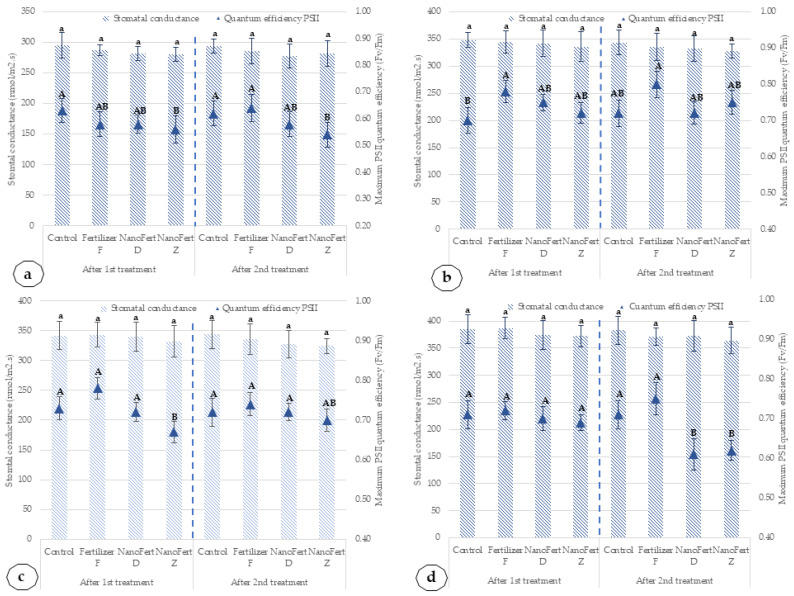
Influence of the application of SNNMs with foliar fertilizer (NanoFert D, NanoFert Z) and foliar fertilizer alone (fertilizer F) on stomatal conductance and maximum photosystem II (PSII) quantum efficiency for apricot cultivars, Amiral and de Valu. (**a**) Amiral cultivar, 2020; (**b**) Amiral cultivar, 2021; (**c**) de Valu cultivar, 2020; (**d**) de Valu cultivar, 2021. The values presented represent means ± standard errors (*n*  =  9 plants). Data sets labeled with different letters within each parameter are significantly different at *p* < 0.05.

The effects of the application of SNNMs with foliar fertilizer (NanoFert D, NanoFert Z) and foliar fertilizer alone (fertilizer F) on stomatal conductance and maximum photosystem II (PSII) quantum efficiency are presented in Figure 7 for apricot cultivars, Amiral and de Valu, during the two experimentation years, 2020 and 2021.

Coverage of the leaves with the film particles formed by zeolites and DE determines a slight reduction in the stomatal conductance and quantum efficiencies of PSII. The decreased quantum efficiency in apricot leaves covered by SNNM particle film formed after foliar spraying for apricot cv. Amiral is 11.1% in 2020 and 7.69% in 2021. In the case of the de Valu cultivar, the decrease is 4.48% and 8.33% in 2020 and 2021, respectively. However, the reduction is not statistically significant in the majority of the situations. In the case of the stomatal conductance, the decrease is much lower, under 1% for all tested combinations, cultivars and years, and it is not statistically significant.

**Figure 8:**
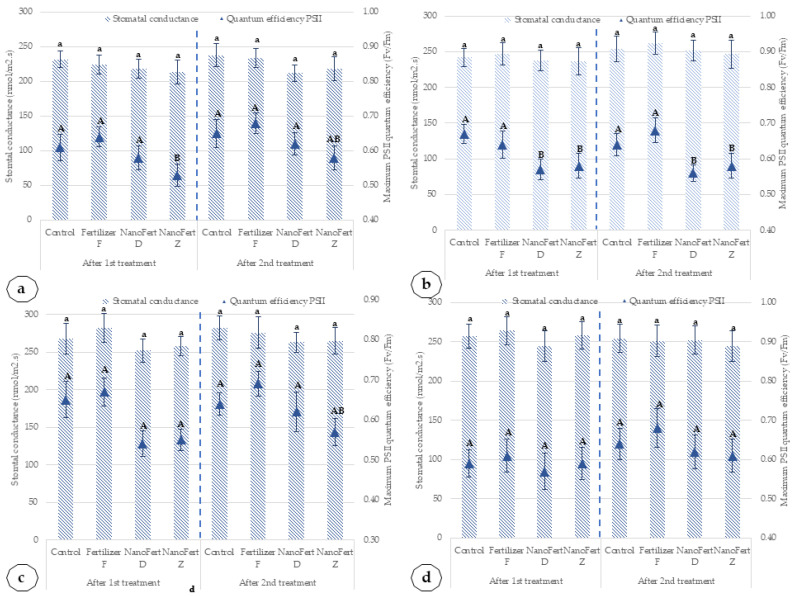
Effects of the application of SNNMs with foliar fertilizer (NanoFert D, NanoFert Z) and foliar fertilizer alone (fertilizer F) on stomatal conductance and maximum photosystem II (PSII) quantum efficiency for peach cultivars, Mimi and Catherine Sel. 1. (**a**) Mimi cultivar, 2020; (**b**) Mimi cultivar, 2021; (**c**) Catherine Sel. 1 cultivar, 2020; (**d**) Catherine Sel. 1 cultivar, 2021. The values presented represent means ± standard errors (*n* = 9 plants). Data sets labeled with different letters within each parameter are significantly different at *p* < 0.05.

The patterns are similar for the two peach cultivars in the two experimental years. Figure 8 presents the effects of the application of SNNMs with foliar fertilizer (NanoFert D, NanoFert Z) and foliar fertilizer alone (fertilizer F) on stomatal conductance and maximum photosystem II (PSII) quantum efficiency for peach cultivars.

In addition, in the peach cultivar, in both experimental years, the application of SNNMs practically did not influence the stomatal conductance and slightly reduced the quantum efficiency of the PSII systems. Compared to untreated control, the most significant reduction of quantum efficiency of the PSII system is for NanoFert Z, applied to cultivar Mimi in 2020—12.30%. In the same year, the maximum reduction of quantum efficiency of the PSII system for Catherine Sel. 1 cultivar was 10.24%, after the application of the NanoFert D product. In 2021, a year with higher precipitation, the effect of the application of SNNMs with foliar fertilizer (NanoFert D, NanoFert Z) and foliar fertilizer alone (fertilizer F) on maximum photosystem II (PSII) quantum efficiency for peach cultivars was statistically significant only for the Mimi cultivar—a reduction of up to 15.38% after the application of NanoFert Z and up to 12.62% after the application of NanoFert D. Such reduction of quantum efficiency in peach after treatment of leaves with film-forming particles was also found for kaolin film. It is probably due to the enhanced reflection of the incident light [48].

**Figure 9:**
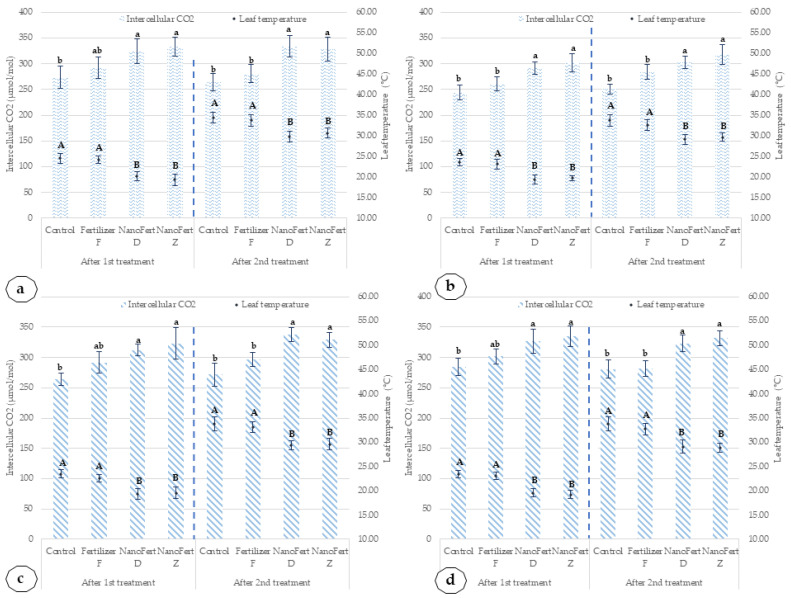
Influence of the application of SNNMs with foliar fertilizer (NanoFert D, NanoFert Z) and foliar fertilizer alone (fertilizer F) on intercellular CO_2_ (Ci) concentrations and leaf temperatures, for apricot cultivars, Amiral and de Valu. (**a**) Amiral cultivar, 2020; (**b**) Amiral cultivar, 2021; (**c**) de Valu cultivar, 2020; (**d**) de Valu cultivar, 2021. The values presented represent means ±standard errors (*n* = 9 plants). Data sets labeled with different letters within each parameter are significantly different at *p* < 0.05.

Other effects specific to particle film formation are reducing leaf temperatures and increasing the intercellular CO_2_ (Ci) concentration. The influence of the application of SNNMs with foliar fertilizer (NanoFert D, NanoFert Z) and foliar fertilizer alone (fertilizer F) on intercellular CO_2_ (Ci) concentrations and leaf temperatures, for apricot cultivars, Amiral and de Valu, is illustrated in Figure 9.

Applying the SNNMs with foliar fertilizer significantly influences the leaves’ temperature and the intercellular CO_2_ (Ci) concentrations. The maximum reduction of leaf temperature for the Amiral cultivar treated with SNNMs is between 4.07 °C and 4.48 °C in 2020 and between 3.72 and 4.30 in 2021. The same pattern is noted for the de Valu cultivar. In 2020, the maximum reduction of leaf temperatures was between 4.15 °C and 4.42 °C. In 2021, the maximum decrease for leaf temperature was between 3.70 °C and 4.25 °C. The year 2021 had higher precipitation and lower temperatures.

**Figure 10:**
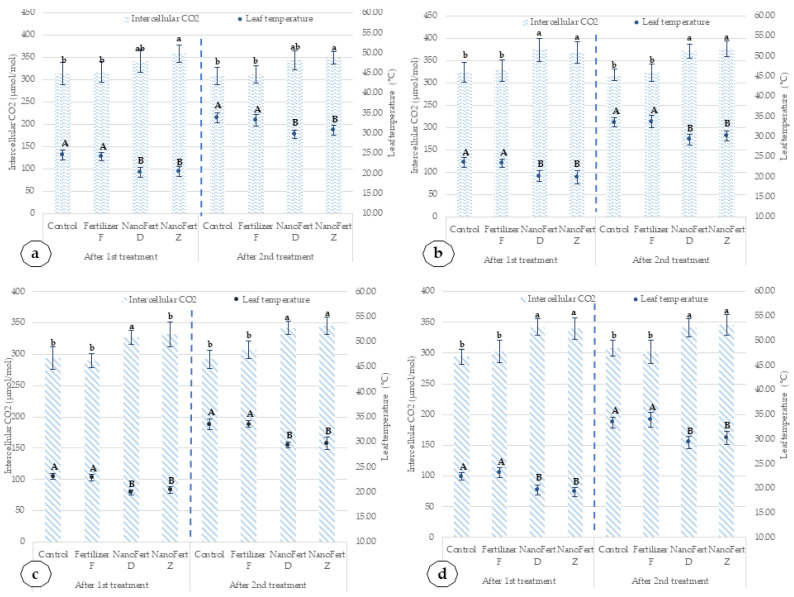
Effects of the application of SNNMs with foliar fertilizer (NanoFert D, NanoFert Z) and foliar fertilizer alone (fertilizer F) on intercellular CO_2_ (Ci) concentrations and leaf temperatures, for peach cultivars, Mimi and Catherine Sel. 1. (**a**) Mimi cultivar, 2020; (**b**) Mimi cultivar, 2021; (**c**) Catherine Sel. 1 cultivar, 2020; (**d**) Catherine Sel. 1 cultivar, 2021. The values presented represent means ± standard errors (*n* = 9 plants). Columns labeled with different letters within each parameter are significantly different at *p* < 0.05.

The intercellular CO_2_ (Ci) concentration in the Amiral cultivar in 2020 is increased up to 27.37% after the first treatment with SNNMs combined with foliar fertilizer and 23.32% after the second treatment with SNNMs combined with foliar fertilizer. In 2021, the increase was 25.50% after the first treatment and 15.77% after the second treatment. In the case of the de Valu cultivar, the increase in Ci is lower, of 22.35% and 15.14% for 2020 and of 16.61% and 17.96% for 2021, respectively. The effect of the application of SNNMs with foliar fertilizer (NanoFert D, NanoFert Z) and foliar fertilizer alone (fertilizer F) on intercellular CO_2_ (Ci) concentrations and leaf temperatures, for peach cultivars, Mimi and Catherine Sel. 1, is presented in Figure 10.

The pattern noted in peach cultivars for leaf physiology parameters is similar to that observed in apricot cultivars cultivated in the same conditions. The leaf temperatures decreased by 3.83 °C and 4.36 °C for the Mimi cultivar, in 2020, after the first and second treatment, respectively. In 2021, for the same cultivar, the decrease was lower—3.48 °C and 4.27 °C, respectively. The reduction of the leaf temperatures for the Catherine Sel. 1 cultivar is smaller. In 2020, the decrease was 3.54 °C and 4.08 °C after the first and second treatment. In 2021, the temperature reduction was also smaller: 3.05 °C after the first treatment and 3.94 °C after the second treatment.

The intercellular CO_2_ (Ci) concentration increases up to 20% in peach cultivars after SNNM and foliar fertilizer treatment. In the Mimi cultivar in 2020, Ci was increased up to 14.01% after the first treatment and by 12.82% after the second treatment. In 2021, the increase was 12.35% and 18.25 after the first treatment and the second treatment, respectively. In the case of the Catherine Sel. 1 cultivar, the increase in Ci was in the same range, of 12.24% and 16.10% for 2020 and of 15.65% and 12.34% for 2021, respectively. The reduction of leaf temperature was up to 4.5 °C during the second application, in a period with high temperatures in the geographical area of RSFG Constanța. Such reduction is significant because a temperature of over 33 °C is close to those that limit the capacity of the first enzyme of the Calvin cycle (dark photosynthesis), ribulose 1·5-bisphosphate carboxylase/oxygenase (Rubisco) and ribulose bisphosphate (RuBP) regeneration [49].

The effects of the application of SNNMs with foliar fertilizer (NanoFert D, NanoFert Z) and foliar fertilizer alone (fertilizer F) on leaf physiological functions are presented in Table 5.

Application of SNNMs and foliar fertilizer decreases the evapotranspiration and increases the net photosynthesis rate. The decrease in evapotranspiration after applying SNNMs with foliar fertilizer ranges from 12.10% to 18.23% in 2020 for apricot cultivars. In 2021, the anti-transpirant effect of the SNNMs with foliar fertilizer on apricot cultivar was lower—E decreased with a maximum of 11.90%.

In 2020, the evapotranspiration reduction in peach leaf cultivars was from 8.54% to 11.78% following treatments with SNNMs. In 2021, for peach cultivars, the pattern was almost similar—a reduction of evapotranspiration of up to 12.05%.

In the meantime, net photosynthesis increases due to the increased intercellular concentration of CO_2_ and reduction of the leaf temperature. In 2020, for the Amiral apricot cultivar, the increase was 21.56% in the case of NanoFert D. For the de Valu cultivar, the increase was 4.86 for NanoFert Z. For the peach cultivars, the increase in net photosynthesis rate was in a shorter range, from 6.72 to 12,47% in 2020 and from 6.34% to 11.92% in 2021. The increase in net photosynthetic rate (Pn) and concomitant reduction of evapotranspiration (E) increased water use efficiency—calculated as Pn over E. In 2020, the maximum increase ranged from 21.42 to 40.85%. In 2021, the increase in WUE was in the range of 19.50–32.70%.

The increased water use efficiency is important for a rain-feed orchard. The yield and main fruit quality indicators of the stone-fruit trees treated with SNNMs and foliar fertilizer are presented in Table 6.

SNNM application together with foliar fertilizer significantly increased the yield of treated stone fruits, apricot and peach. In the Amiral apricot cultivar, this effect on yield is more evident in 2020, a year with low precipitation. The yield increase is significant, with 14.03% in the case of NanoFert D and 1.65 in the case of NanoFert Z. In 2021, the increase in the yield after treatment with SNNMs and foliar fertilizers is less than 10% for both treatments—8.63% for NanoFert D and 9,61% for NanoFert Z. The yield increase after treatment is still significant. It is lower because the yield of non-treated control was significantly higher, of 8.70%, in 2021 compared to 2020. The increase in the yield for the Amiral cultivar from one year with low precipitation to another year with higher rainfall is more significant than for the other tested cultivars, apricot (de Valu) or peach (Mimi, Catherine Sel. 1). For these, the yield increase for non-treated control is around 5–105.56% for the production of the de Valu cultivar in control 2021 compared to control 2020, 104.41% in the case of the Mimi cultivar and 105.23% for the Catherine Sel. 1 cultivar. The lower reaction of these cultivar breeds in non-irrigated conditions to the higher rainfall is also related to a more equilibrated response of these cultivars to the applied treatments. In the case of the de Valu apricot cultivar, the application of foliar fertilizer alone determined a significant yield increase of around 6–5.74% in 2020 and 6.20% in 2021. Additional application of SNNMs and foliar fertilizer determined an increase in the production by 7.61% in the case of NanoFert D and by 6.80% in the case of NanoFert Z. In 2021, an additional increase was 6.25% for NanoFert D and 4.87% for NanoFert Z.

In the case of the apricot cultivar, the effects are similar. In 2020, the yield increase was around 4.50% in the case of applying foliar fertilizer alone. The total increase ranged from 10.58% to 14.17% in the case of NanoFert D and NanoFert Z. In 2021, a year with a higher precipitation rate, the increase in the yield determined by the application of foliar fertilizer alone was around 7%. The total increase after the application of foliar fertilizer together with SNNMs ranged from 12.82% to 16.36%.

A possible explanation for the better response of the treated stone-fruit trees to applying foliar fertilizer in 2021 is related to the role of water in solute (including mineral nutrients) absorption into the leaves [50].

The effect of the application of foliar fertilizers and SNNMs on total soluble solids (TSS, %) and titratable acidity (A, mg equiv. malic acid) is less obvious and depends on the species and cultivar. Apricots tend to accumulate more acid, and de Valu is a cultivar where a high level of organic acid balances the sweetness of the fruit. Peach cultivars accumulate more TSS and produce fewer organic acids. In general, applying SNNMs and foliar fertilizer slightly enhances TSS accumulation (at the limit of statistical differences) and has a limited impact on titratable acidity.

**Figure 11:**
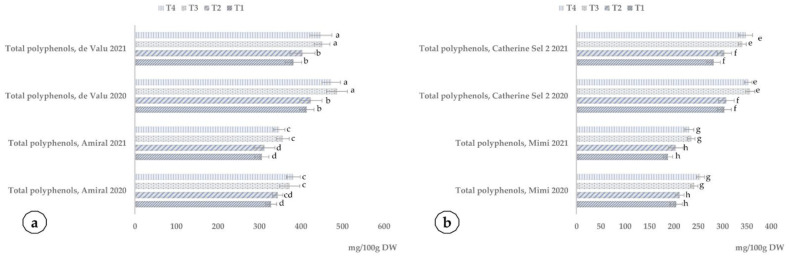
Influence of the application of SNNMs with foliar fertilizer (T3, NanoFert D; T4, NanoFert Z) and foliar fertilizer alone (T2) compared to control (T1) on the accumulation of polyphenols in apricot (**a**) and peach cultivars (**b**). The values presented represent means ± standard errors (*n* = 9 fruits). Columns labeled with different letters are significantly different at *p* < 0.05.

The significant effect of SNNM application is on the accumulation of polyphenols and flavonoids in fruits—Figure 11.

The increase in polyphenol accumulation of apricot cultivars after treatment with SNNMs and foliar fertilizer ranges between 12.63 and 18.28% and is statistically significant in each year of testing. In addition, in peach, the treatment with SNNMs and foliar fertilizer determines an enhanced accumulation of polyphenols—from 14.15 to 24.40%.

## 3. Discussion

Information related to SNNMs from Romanian quarries, natural zeolites from Rupea quarries and diatomaceous earth from the Pătârlagele quarry, is relatively scarce. These siliceous natural nanomaterials were successfully used to remove Mn and Fe ions from a binary solution—natural zeolites from Rupea [51]—or for stored grain protection—DE from Pătârlagele [52,53]. Such applications are proof of superior sorption active surfaces and cation exchange capacity. However, according to our knowledge, investigations of the morphology and ultrastructure of these SNNMs or determination of the characteristics related to their agricultural applications, such as BET active surface or cation exchange capacity, were not yet conducted. Our findings demonstrate that the chosen SNNMs from Romanian quarries have features similar to those of other natural materials used for plant treatment, natural zeolites [54] or diatomaceous earth [55]. The nanoporous structure of these natural materials, with active surface binding hydrophilic molecules and cations, make them suitable as carriers for agrochemicals [56].

Our work goal was to test SNNMs’ double function: as a (fine) powder for particle film formation and as an active carrier for foliar fertilizer. The results from our experiments confirm the film formation on the surface of the treated leaves. Particle film formation slightly decreases the quantum efficiency of PSII and stomatal conductance. Coverage of the leaves with SNNMs reflects a part of the incident light and reduces the accessibility of stomata. At the same time, particle film formation reduces leaf temperature and increases intercellular CO_2_ (Ci). Such effects are specific for particle film formation and were described for kaolin [48] on stone fruits from an area with Mediterranean conditions. Zeolites are known to produce particle film on fruit tree leaves [28]. However, according to our knowledge, particle film formed by diatomaceous earth was not yet reported. Our study proved that it is also possible to use DE for particle film formation. Overall, the efficiency of DE in generating effects specific to particle film formation on the leaves was almost the same with natural zeolites.

Coverage of the leaves with dust (cement or soil) reduces light availability and affects CO_2_ fixation [57]. However, in the case of particle film formed by zeolites or diatomaceous earth, CO_2_ fixation and water use efficiency are not reduced. Reduced evapotranspiration compensates for the slightly reduced performance of the light phase of photosynthesis. In the case of zeolites, the local concentration of carbon dioxide due to CO_2_ fixation in the zeolite nanostructure further promotes dark-phase photosynthesis [31]. In our experiments, DE proved to have a similar effect with zeolites. DE is a material that also has CO_2_ adsorption characteristics [58].

The effects on yields and fruit quality support the working hypothesis of SNNMs as active carriers for foliar fertilizers. Zeolites were used to produce fertilizer nano-composites that slow-release the nutrients into the soil [59]. Zeolites’ nanoporous structure generates a sizeable active surface and a significant cation exchange capacity. Clinoptilolite was used as a carrier of nitrogen fertilizer with controlled release in the soil [60]. Natural zeolites were proven to optimize nitrogen utilization and nitrogen accumulation from the soil in the plant tissue [61]. Zeolites’ effects on utilization efficiency are not related only to nitrogen. Zeolite carriers were shown to improve the utilization efficiency of various other mineral nutrients, macro-nutrients, e.g., potassium [40], oligo-nutrients, e.g., zinc [41], and micro-nutrients, e.g., manganese [42]. The slow-release effect of zeolites is not related only to cationic mineral nutrients. Silicate also interacts with phosphates, slow-released from phosphatic fertilizer. Zeolites were demonstrated to improve corn yield and reduce phosphate leaching [43]. Clinoptilolite improves phosphorus availability in acidic soil [62]. Zeolites and rock phosphate were shown to be efficient fertilizers for potted plants [63]. Overall, the application of zeolites to various soil types was demonstrated to reduce leaching and enhance nutrient uptake and nutrient use efficiency [64]. Zeolites, especially as “nutrients-augmented zeolites,” are an illustrative example of “nanomaterial-enhanced fertilizers” [65]. Overall, zeolites improve fertilizers’ application eco-efficiency because they reduce nutrient loss and negative environmental impact [59].

Porous nanomaterial was described recently as the ”main vein of agricultural nanotechnology” [56]. Despite its nanoporous nature, diatomaceous earth was less considered as a nano-carrier for the fertilizers. Spent filtration DE from the brewery was proposed as a carrier for pelletized fertilizers with macro-nutrients [66]. Calcined DE was demonstrated to reduce nitrate leaching from cropland [67].

Despite this well-known ability to act as a slow-releasing carrier for mineral nutrients in the soil, the natural zeolites and diatomaceous earth were not yet used as a carrier for foliar fertilizer. A formulation was developed based on zeolites and copper, which was used to control grape downy mildew [68] and grapevine gray mold and sour rot [54] and not for the nutrition of the cultivated plants.

The final effects of the simultaneous application of natural zeolites (clinoptilolites) from the Rupea quarry and diatomaceous earth from the Pătârlagele quarry, together with a foliar fertilizer, were the increased yield and fruit quality of the treated stone-fruit trees.

Increased crop quality was also reported for other cultivated plants treated with zeolites. Oils obtained from olives treated with chabazite zeolites have superior olfactory and gustatory qualities [40]. Chabazite zeolites enhance the accumulation of the anthocyanins in *Vitis vinifera* cv. Sangiovese [41]. There were proposed two mechanisms for this crop quality increase. One is related to the decrease in the leaf temperature and concomitant stress reduction. The other one is related to a putative elicitor of secondary metabolism released from the SNNM particle film.

Soluble silicon species could be such an elicitor of secondary metabolism. Soluble silicon is a plant biostimulant [69], its beneficial effects being related to cultivated plant protection against biotic and abiotic stress [70]. Silicon application was demonstrated to improve strawberry quality [71]. Diatomaceous earth is a proven source of soluble silicon species [1]. Amorphous DE biosilica is precipitated after the polycondensation of the silicic acid from the “soluble silicon pool,” a stabilized H_4_SIO_4_ solution formed between diatoms’ external membranes and cell wall [72]. The polycondensation of the silicic acid from the “soluble silicon pool” under the effects of molecules associated with diatom cell walls occurs at a lower concentration than that from chemical/not biocatalysed reactions. Therefore, the resulting polysicilic polymer, the amorphous biosilica, SiO_2_xnH_2_O, is in a metastable state and rapidly releases silicic acid in an aqueous environment [73]. Due to this characteristic, DE-based products were used to provide soluble silicon species, i.e., silicic acid and its dimers and trimers, to the plants by treatment in the rhizosphere [74,75,76] and phyllosphere [77]. Zeolites also release soluble silicon species. It was demonstrated that Si-rich mineral zeolite protects barley plants against cadmium stress [10].

One of the effects specific to plant biostimulants/soluble silicon is the enhancement/benefit of nutrient uptake. DE applied with standard fertilizer increases rice yield [74]. A DE-based product, Agrisilica^®^ (Agripower, Sydney, Australia), prepared from *Melosira granulate* frustules, increased the yield of watermelon [78], sugar beet [79] and sugarcane [80]. Another plant biostimulant effect/silicon effect increases plant resistance to abiotic stress, such as water stress or heavy metal stress. A commercial DE product (Perma-Guard, Inc., Kamas, UT, USA), used as a soluble silicon supplement to the growing medium, increased the tolerance to water stress of potted ornamentals—black-eyed Susan, *Rudbeckia hirta*, dahlia, *Dahlia Cav. × hybrid*, and daisy, *Gerbera jamesonii* [76].

SNNMs applied as foliar treatment in stone-fruit trees, apricot and peach, demonstrate effects specific to plant biostimulants: increased nutrient use efficiency (in a direct relationship with enhanced dark photosynthesis), plant protection against heat and drought stress and improvement of the fruit quality. Further demonstration of the mechanism of action involved in such effects probably will decide the potential inclusion of SNNMs in the category of plant biostimulants. The release of the soluble silicon species is an argument for inclusion in the plant biostimulant category. Physical mechanisms related to particle film formation and anti-transpirant and temperature reduction effects are an argument to consider SNNMs with other particle-film-forming compounds, such as kaolin. Most probably, both mechanisms are involved.

Diatomaceous earth was not yet applied to generate a particle film on the leaves of cultivated plants. DE application with foliar fertilizer determines effects similar to other particle-film-generating materials—i.e., zeolites and kaolin. Further studies are necessary to optimize DE application as an anti-transpirant and heat protectant.

Application of the foliar fertilizer together with natural zeolites or diatomaceous earth represents a strategy to increase the foliar fertilizer’s efficiency and reduce the impact of biotic and abiotic stress on treated plants. SNNMs act as a slow-release carrier for mineral nutrients applied to leaves. The lower temperature and the water retention on leaves promote foliar penetration of mineral nutrients through aqueous channels from the cuticles. Particle film from leaves reduces heat stress and evapotranspiration. The particle film formation was demonstrated to reduce pathogens and arthropods’ attacks on leaves.

## 4. Materials and Methods

### 4.1. Plant Material and Experimental Site

The plant material was represented by two Romanian cultivars of apricot, *P. armeniaca*, Amiral, and de Valu, and two Romanian cultivars of peach, *P. persica*, Mimi, and Catherine Sel. 1. The used Romanian cultivars are breeds from the Research Station for Fruit Growing (RSFG) Constanța. Amiral is an early ripening cultivar, with sweets and aromatic fruits of oblong shape and orange and red-carmine skin, usually consumed fresh. The apricot cultivar de Valu is a mid-term ripening cultivar, with large sweets and aromatic fruits, having pale orange and red-orange skin, consumed fresh and after industrialization. Both peach cultivars are middle-term ripening cultivars, with large fruits, sweet and aromatic, having yellow-orange skin. Mimi is a freestone fruit for fresh consumption. Catherine Sel. 1 is a clingstone peach used for industrialization, jams and compotes. The used rootstocks were the wild apricot (*P. armeniaca*, var. *minor*) for apricot cultivars and hybrid Tomis 1 (*P. persica*) for peach cultivars.

The experiments were performed in 2020 and 2021 in the orchard of RSFG Constanța (Figure S1 in the Supplementary Materials). This orchard is located in southeastern Romania, Dobrogea, and it is a non-irrigated/rain-feed orchard. The geographical coordinates were the following: apricot, cv. Amiral, 44°10′41.47″ North latitude, 28°29′5.12″ East longitude; apricot, de Valu cv., 44°10′40.76″ North latitude, 28°29′5.43″ East longitude; peach, cv. Mimi 44°10′38.27″ North latitude, 28°29′4.21″ East longitude; Catherine Sel. 1 44°10′38.05″ North latitude, 28°29′4.54″ East longitude. The altitude of the orchard is 30 m. The RSFG Constanța orchard is established on calcar chernozem soil (32.8% sand, 34.3% silt and 32.9% clay), formed on greenschist parental rocks. The soil has a pH of 8.1 (in water) and a humus content of 2.76% in the upper (0–20 cm) soil horizon. The average values of multi-annual (1975–2015) temperature, total precipitation, wind speed and sunshine daily duration for the experimental site RDSFG Constanța are 11.8 °C, 497.3 mm, 4.4 m s^−1^ and 7.2 h, respectively. The average monthly temperature and precipitation during the experimentation period 2020 and 2021 are presented in Table 7, compared to average multi-annual temperature and precipitation.

During the experimentation period, 2020 and 2021, the climatic conditions were characterized by a very different rainfall pattern. In 2020, the total rainfall from March to August was 98 mm, significantly lower than the multi-annual average of 267.5 mm for the same period. The total precipitation for 2020 was 370 mm, lower than the multi-annual average. In 2021, the total rainfall in March–August was 405.7 mm, significantly higher than the multi-annual average. From January until August 2021, the total precipitation reached the multi-annual average—497.7 mm.

Trees were planted in 2011, being nine and ten years old during the experimentation period. The trees were planted at 4/4 m (625 trees/ha) and trained in vessel shape. During the winter of 2020 and 2021, the orchard was fertilized with 100 kg ha^−1^ of urea, Ca(H_2_PO_4_) and K_2_SO4. The plant protection treatments of the orchard were conducted according to the area’s recommended agricultural best practices and are detailed in Tables S1 and S2 (from the Supplementary Materials).

### 4.2. Siliceous Natural Nanomaterial Preparation and Characterization

Natural zeolites from Rupea quarry (Zeolites Production, Rupea, Brașov) and diatomaceous earth from Pătârlagele quarry (Industriile de Diatomit, Pătârlagele, Buzău, Romania) were used. Natural zeolites of Rupea quarry are of clinoptilolite type—around 85% is clinoptilolite. The elemental composition of the thermally activated Rupea natural zeolite is: 61.2% O; 24.0% Si; 5.1% Al; 1.8% Ca; 2.2% K; 0.8% Fe; 0.7% Mg; 0.6% Na [51]. The DE from the Pătârlagele quarry was formed predominantly in Oligocene by marine diatoms. The main component of Pătârlagele DE is represented by diatom frustules, with a median dimension of 5,4 µm. The total silica content is around 85%, with 81.2–84.1 amorphous SiO_2_ (opal-A) and less than 0,9% crystalline SiO_2_/quartz. Other minerals present in DE from Pătârlagele are clay, illite and kaolinite [52,81].

The preparation process of the two selected SNNMs was the following: porous rock from the quarries was crushed (in Jaw Crusher BB 50, Retsch-Verder Scientific, Haan, Germany), washed with pure water at room temperature, dried at 105 °C for 24 h (in an ED 115 drying chamber, Binder, Tuttlingen, Germany) and milled in a planetary ball mill (PM 100, Retsch-Verder Scientific, Haan, Germany, with agate grinding jar and agate balls) to obtain particles sized 1–2 mm and activated. The activation of zeolites was performed by thermal treatment, at 200 °C for 2 h, as the first stage, followed by second activation treatment at 250 °C for 1 h [51]. The activation of DE was performed by mixing with 0.1 M HCl for 2 h at room temperature, in a ratio of 1 g DE to 10 mL HCl, followed by washing with 10 mL NaOH 0.1 M and 20 mL of pure water and drying at 105 °C for 24 h.

The SNNMs were characterized by FTIR spectroscopy, X-ray diffraction, scanning electron microscopy, transmission electron microscopy and liquid nitrogen porosimetry. The FTIR spectra of the SNNMs samples were obtained on KBr pellets with 1% of each sample, using a Fourier transform infrared (FTIR) spectrophotometer (IRTracer-100, Shimadzu, Kyoto, Japan), with a resolution at 0.25 cm^−1^ and scanning number of 20 spectra/second, in ATR mode, for the interval 4000–400 cm^−1^. The crystallinity of the powdered SNNM samples was characterized by using a SmartLab diffractometer (Rigaku, Tokyo, Japan), emitting a CuK_α1_ (λ = 1.54059Å) radiation. The morphology and surface texture of the SNNM samples were observed using scanning electron microscopy (ESEM FEI Quanta 200, FEI, Hillsboro, OR, USA), including an energy-dispersive X-ray spectroscopy (EDX) detector. EDX was used to determine the elemental composition of SEM-analyzed SNNM samples. Ultrastructure of SNNM samples was characterized by transmission electron microscopy (Tecnai™ G2 F20 TWIN Cryo-TEM, FEI, Hillsboro). Brunauer–Emmet–Teller (BET) analysis of the surface area, total pore volume and average pore diameter was performed in a NOVA 2200e system (Quantachrome, Boynton Beach, FL, USA). BET analysis was performed for three samples, resulting from different batches of SNNMs extracted from Romanian quarries.

The cation exchange capacity was determined according to the method described by Cerri et al. [82]. Briefly, the SNNM material, DE or natural zeolites, was dispersed in 0.5 M NH_4_Cl solution, in a ratio of 3 g to 100 mL solution, at 65 °C. Five successive exchange cycles were performed at 30 min; 30 min; 1 h; 2 h; 2 h. The concentration of exchangeable cations was determined by atomic absorption spectrophotometry (ICE 3300, Thermo Fisher, Waltham, MA, USA). The sum of the released cations was determined and considered as cation exchange capacity (CEC). NH_4_Cl was purchased from Sigma-Aldrich (Merck Group, Darmstadt, Germany) and was of 99.5% purity. The cation exchange capacity was determined for three samples, resulting from different batches of SNNMs extracted from Romanian quarries

### 4.3. Preparation and Analysis of Foliar Fertilizer with SNNMs

**Figure 12:**
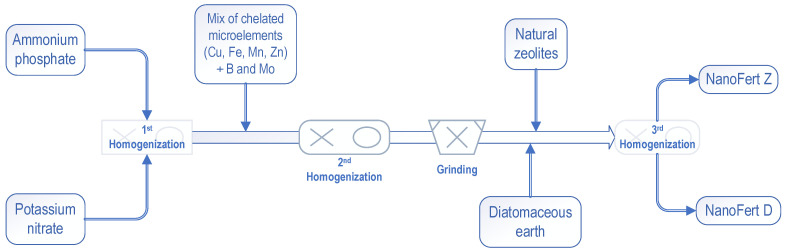
Illustration of the preparation process of NanoFert Z (with natural zeolites) and NanoFert D (with diatomaceous earth) foliar fertilizers.

Two fertilizers with activated SNNMs were prepared, NanoFert Z (with zeolites) and NanoFert D (with diatomaceous earth). The foliar fertilizers were NPK 7:30:4, with micro-elements. The preparation process is illustrated in Figure 12.

The preparation process consists of the following steps: weighing the soluble microelement sources, ammonium phosphate and potassium nitrate and 1st homogenization for 15 min; dosage of the mix of chelated elements (with EDTA—Cu, Fe, Mn, Zn) plus Boron (B) and Molybdenum (Mo), followed by second homogenization for 15 min and grinding; dosage of the activated diatomaceous earth or activated natural zeolites and 3rd homogenization for 30 min. The equipment used was an overhead shaker-mixer Reax 20 (Heidolph, Schwabach, Germany) and a PM20 planetary ball mill (PM 100, Retsch-Verder Scientific, Haan, Germany, with an agate grinding jar and agate balls). The proportion of mixing SNNMs and foliar fertilizer was 3 kg of natural zeolites to 1 kg of foliar fertilizer mixture and 3 kg of diatomaceous earth to 1 kg of foliar fertilizer. The used raw materials for NPK ingredients of the foliar fertilizer were: ammonium phosphate (EuroChem, Zug, Switzerland), technical grade, 99.5% purity, 0.073% humidity, ammonia nitrogen 12.1%, phosphorus (P_2_O_5_) 61,0%, and potassium nitrate (SQM Europe, Antwerp, Belgium), technical grade, purity 99%, nitrate-nitrogen 13,8%, potassium (K_2_O) 46,4%. The mix of chelates (EDTA) of the microelements (Cu, Fe, Mn, Zn) plus B and Mo was supplied by Solarex (Craiova, Romania) and had 99,8% purity, with Cu 0.71%, Fe 6.41%, Mn 3.20%, Zn 0.96%, B 1.28% and Mo 4.48%.

The resulting fertilizer formulation was analyzed according to standard methods—EU Regulation 2003/2003, Annex IVB. Total nitrogen was determined according to the EN SR EN 15476/2009 method, which involves reducing Devarda’s alloy, ammonia distillation and titration of the excess sulfuric acid (used for capturing ammonia) in the presence of the neutral red indicator. Phosphorus (P) was determined after extraction by gravimetric determination according to ISO 6598:1996 method. Potassium (K) was determined according to EN 15477:2009 method. The determination of the copper, iron, manganese and zinc was performed according to EN 16965/2018 by flame atomic absorption spectrometry (FAAS), using an ICE 3300 atomic absorption spectrometer (Thermo Fisher Scientific, Waltham). Boron was determined according to EN 17041/2018 standard, using spectrometry with azomethine-H. The analyses of fertilizers were performed in a laboratory certified according to ISO 17025. The calibration curves for the microelements were performed with standard reference material (Certipur^®^ ICP, Merck Group, Darmstadt).

### 4.4. Application of Foliar Fertilizer with SNNMs

The following treatments were applied: T_1_—control, sprayed with water; T_2_—foliar fertilizer, concentration 0.4% (equiv. to 5 kg/ha foliar fertilizer NPK 7:30:4, with micro-elements); T_3_—foliar fertilizer NanoFert Z concentration 1.6% (equivalent to 5 kg/ha foliar fertilizer and 15 kg/ha Rupea natural zeolites); T4—foliar fertilizer NanoFert D, concentration 1.6% (equivalent to 5 kg/ha foliar fertilizer and 15 kg/ha diatomaceous earth). The experiments were performed with a completely randomized block design, using four fertilization treatments and four replications per treatment and year (each replicate was conducted with three individual trees). Two treatments were applied each year, at phenological stage 73, second fruit fall and 78, fruit about 80% of final size, according to the BBCH scale [83,84]. The treatments were applied with a backpack mistblower (SR 420, Stihl, Waiblingen, Germany). The volume applied for each fruit tree was 2 L, corresponding to a normalized spraying volume per ha of 1250 L. Treatments were applied in the morning, between 7.30 and 10.30, at a temperature lower than 24 °C.

### 4.5. Determination of Physiological Characteristics of Apricot and Peach Plants

Apricot and peach plants were monitored one week after the first treatment and one week after the second. The physiological parameters of treated stone-fruit trees were measured, i.e., stomatal conductance, fluorescence and leaf physiological functions. The leaves’ stomatal conductance (nmol m^−2^ s^−1^) was measured with a Delta T AP4 porometer (Delta-T Devices, Burwell, UK) in four leaves per tree from each treatment replicate. The measurements were conducted in the morning. The determinations of the chlorophyll fluorescence of leaves were performed with a PAM fluorometer (Walz PAM 2500, Effertlich, Germany), according to manufacturer’s instructions. The measurements were conducted on 10 randomly chosen leaves per replicate (selected from representative, healthy plant upper leaves from each repetition). Before starting the determination, the leaves were pre-darkened for 60 min. using a brown paper bag. The saturation light pulses were applied on pre-darkened leaves. Maximum photosystem II (PSII) quantum efficiency was determined as the ratio between F*v*, variable fluorescence, and F*m*, the maximum fluorescent yield in the dark-adapted state. The leaf physiological function was determined using a leaf gas-exchange unit (model LCpro T, ADC BioScientific Ltd., Herts, UK). The unit was used with a broadleaf chamber, with a window area of 6.25 cm^2^, operated at a 300–350 mL/min flow rate. The leaf gas-exchange unit was used to determine or calculate leaf temperature, evapotranspiration (E) and net photosynthetic (Pn) rates, intercellular CO2 (Ci) and water use efficiency (WUE, as Pn over E). Each determination was repeated three times.

### 4.6. Determination of Yield and Fruit Quality

Fruits were harvested at maturity from each tree and weighed on an electronic precision balance (model ME, Mettler-Toledo, Columbus, OH, USA). The main characteristics of fruit quality, total soluble solids and acidity were determined per each replicate. Five ripe fruits from each replicate were hand-peeled and then squeezed by using a commercial juicer. The resulting juice was centrifuged for 20 min at 4 °C and 10,000× *g* (Universal 320 R centrifuge, Hettich, Tuttlingen, Germany). The total soluble solids were assayed with a digital refractometer (RX 5000α, Atago, Tokyo, Japan). The juice’s acidity was titrated with a solution of 0.1 N NaOH until pH 8.1 (Titrino 848 Plus, Metrohm, Herisau, Switzerland) and expressed as % malic acid. The total polyphenols from the clarified juice were determined by Folin–Ciocâlteu method [85], with some modifications [86]. Briefly, to 150 µL of the sample were added 750 µL of Folin–Ciocâlteu reagent, 4 mL of 15% Na_2_CO_3_ and distilled water, until a final volume of 15 mL. After 2 h of incubation at room temperature, the optical density at λ = 756 nm was measured. The total phenolic compounds were expressed as gallic acid (GA) equivalents, based on a calibration curve constructed with known concentrations of gallic acid. All of the determinations were conducted in triplicate. All of the reagents used were analytical-grade reagents purchased from Sigma-Aldrich (Merck Group, Darmstadt).

### 4.7. Statistical Analysis

In this work, the standard uncertainty of foliar fertilizers and SNNM foliar fertilizers was calculated as standard deviation by assuming a rectangular distribution. The data from the experiments on stone-fruit trees (apricot and peach) were statistically analyzed by analysis of variance (ANOVA), using the SPSS 21 software package (IBM, Armonk, NY, USA). The least significant difference (LSD) test separated treatment means within each measured parameter, at a significance level of *p* < 0.05.

## 5. Conclusions

Our study demonstrated that natural materials with a nanoporous structure, zeolites (clinoptilolites) and diatomaceous earth, are efficient (nano)carriers for foliar fertilizers. The overall effects on tested stone-fruit trees include effects specific to particle film formed on leaves. Diatomaceous earth, with large and small nanopores and a network of nanotunnels/nanochannels, was also proven for the first time to determine an impact specific for particle film formation on the leaves of apricots and peaches.

## Figures and Tables

**Table 1:** Total pore volume and average pore diameter of the natural zeolites from Rupea quarry and diatomaceous earth from Pătârlagele quarry.

Sample	Total Volume of the Pores (cm^3^ g^−1^)	The Average Diameter of the Pores (nm)
Natural zeolites, before activation	0.0578 ± 0.0027	5.339 ± 0.347
Natural zeolites, after activation	0.0608 ± 0.0032	5.517 ± 0.383
Diatomaceous earth, before activation	0.0692 ± 0.0042	8.919 ± 0.0483
Diatomaceous earth, after activation	0.0754 ± 0.0038	9.127 ± 0.417

**Table 2:** Composition of the prepared foliar fertilizer F1, estimated value (according to receipt) and determined value.

Analyte	Unit	Estimated Value	Determined Value	Uncertainty
Nitrogen (N total)	%	3.5	3.57	±0.12
Phosphorus (as P_2_O_5_)	%	15	15.62	±1.14
Potassium (as K_2_O)	%	2	2.41	±0.20
Copper (Cu)	%	0.002	0.0032	±0.0003
Zinc (Zn)	%	0.0025	0.0024	±0.0004
Iron (Fe)	%	0.017	0.0159	±0.0016
Manganese (Mn)	%	0.008	0.0076	±0.0006
Boron (B)	%	0.003	0.0035	±0.0004

**Table 3:** Composition of the prepared foliar fertilizer, NanoFert D, estimated value (according to receipt) and determined value.

Analyte	Unit	Estimated Value	Determined Value	Uncertainty
Nitrogen (N total)	%	3.5	3.78	±0.11
Phosphorus (as P_2_O_5_)	%	15	15.38	±1.31
Potassium (as K_2_O)	%	2	2.23	±0.25
Copper (Cu)	%	0.002	0.0028	±0.0003
Zinc (Zn)	%	0.0025	0.0028	±0.0004
Iron (Fe)	%	0.017	0.0172	±0.0016
Manganese (Mn)	%	0.008	0.0084	±0.0006
Boron (B)	%	0.003	0.0029	±0.0004

**Table 4:** Composition of the prepared foliar fertilizer, NanoFert Z, estimated value (according to receipt) and determined value.

Analyte	Unit	Estimated Value	Determined Value	Uncertainty
Nitrogen (N total)	%	3.5	3.54	±0.13
Phosphorus (as P_2_O_5_)	%	15	15.87	±1.20
Potassium (as K_2_O)	%	2	1.89	±0.17
Copper (Cu)	%	0.002	0.0024	±0.0003
Zinc (Zn)	%	0.0025	0.0032	±0.0004
Iron (Fe)	%	0.017	0.0182	±0.0013
Manganese (Mn)	%	0.008	0.0090	±0.0010
Boron (B)	%	0.003	0.0033	±0.0004

**Table 5:** The effects of the application of SNNMs with foliar fertilizer (NanoFert D, NanoFert Z) and foliar fertilizer alone (fertilizer F) on leaf physiological functions, evapotranspiration (E) rate (E, mmol m^−2^ s^−1^), net photosynthetic (Pn) rate, (Pn, µmol m^−2^ s^−1^) and water use efficiency (WUE, as Pn over E, mmol.mol^−1^) of apricot and peach tested cultivars.

Cultivar	Treatment	2020			2021		
		E	Pn	WUE	E	Pn	WUE
Amiral	T1, control, 1st treatment	4.52 a	12.57 b	2.78 c	5.46 a	13.43 b	2.46 c
	T2, fertilizer F, 1st treatment	4.41 a	12.83 b	2.91 c	5.02 a	14.88 a	2.96 c
	T3, NanoFert D, 1st treatment	3.84 b	15.28 a	3.98 a	4.92 a	15.37 a	3.12 bc
	T4, NanoFert Z, 1st treatment	3.92 b	14.74 a	3.76 a	4.81 a	15.14 a	3.15 bc
Amiral	T1, control, 2nd treatment	4.29 a	11.82 b	2.75 c	5.19 a	12.76 bc	2.48 c
	T2, fertilizer F, 2nd treatment	4.23 a	11.93 b	2.82 c	4.67 a	13.91 b	2.98 c
	T3, NanoFert D, 2nd treatment	3.53 b	14.06 a	3.94 a	4.53 ab	14.32 a	3.16 bc
	T4, NanoFert Z, 2nd treatment	3.68 b	13.71 a	3.72 a	4.69 a	14.19 ab	3.08 bc
de Valu	T1, control, 1st treatment	2.74 c	8.33 e	3.04 c	3.81 c	10.16 d	2.67 c
	T2, fertilizer F, 1st treatment	2.71 c	8.25 e	3.05 c	3.28 d	10.71 d	3.26 bc
	T3, NanoFert D, 1st treatment	2.23 d	8.62 e	3.87 a	3.32 d	11.73 c	3.53 b
	T4, NanoFert Z, 1st treatment	2.32 d	8.76 e	3.78 a	3.05 d	11.64 c	3.83 b
de Valu	T1, control, 2nd treatment	2.94 c	8.71 e	2.96 c	3.74 c	10.27 d	2.75 c
	T2, fertilizer F, 2nd treatment	2.88 c	8.64 e	3.00 c	3.53 cd	11.42 c	3.24 bc
	T3, NanoFert D, 2nd treatment	2.37 d	9.12 de	3.85 a	3.42 cd	12.83 b	3.75 a
	T4, NanoFert Z, 2nd treatment	2.44 d	8.82 e	3.61 a	3.35 cd	12.76 b	3.81 a
Mimi	T1, control, 1st treatment	3.82 b	11.15 c	2.90 c	4.68 a	12.72 bc	2.72 c
	T2, fertilizer F, 1st treatment	3.66 b	12.27 b	3.35 b	4.79 a	13.24 b	2.76 c
	T3, NanoFert D, 1st treatment	3.14 c	12.62 b	4.02 a	4.16 bc	14.12 ab	3.39 b
	T4, NanoFert Z, 1st treatment	3.07 c	12.54 b	4.08 a	4.24 b	14.27 b	3.37 b
Mimi	T1, control, 2nd treatment	3.53 b	10.50 c	2.97 c	4.40 b	11.95 c	2.72 c
	T2, fertilizer F, 2nd treatment	3.37 bc	11.41 b	3.39 b	4.45 b	12.62 bc	2.83 c
	T3, NanoFert D, 2nd treatment	2.86 c	11.61 b	4.06 a	3.83 c	13.44 b	3.51 b
	T4, NanoFert Z, 2nd treatment	2.82 c	11.54 b	4.08 a	4.07 bc	13.70 b	3.37 bc
Catherine Sel. 1	T1, control, 1st treatment	3.30 bc	9.03 de	2.74 c	4.34 b	11.33 c	2.61 c
	T2, fertilizer F, 1st treatment	3.09 c	9.73 d	3.15 b	4.14 bc	10.99 cd	2.64 c
	T3, NanoFert D, 1st treatment	2.81 c	10.01 d	3.57 b	3.78 c	11.49 c	3.04 c
	T4, NanoFert Z, 1st treatment	2.80 c	9.88 d	3.53 b	3.72 c	11.75 c	3.16 bc
Catherine Sel. 1	T1, control, 2nd treatment	3.47 b	9.82 d	2.83 c	4.57 b	11.68 c	2.56 c
	T2, fertilizer F, 2nd treatment	3.32 bc	10.24 cd	3.08 c	4.43 b	11.82 c	2.67 c
	T3, NanoFert D, 2nd treatment	3.05 c	10.48 c	3.44 b	4.06 bc	12.42 bc	3.06 bc
	T4, NanoFert Z, 2nd treatment	2.98 c	10.62 c	3.56 b	3.96 c	12.58 bc	3.18 bc

Values labeled with different letters within a series are significantly different at *p* < 0.05. The data comparison was performed within the same cultivar each year.

**Table 6:** The influence of the application of SNNMs with foliar fertilizer (NanoFert D, NanoFert Z) and foliar fertilizer alone (fertilizer F) on yield (kg/ha) and main quality indicators, total soluble solids (TSS, %) and titratable acidity (A, mg equiv. malic acid) of apricot and peach tested cultivars.

Cultivar	Treatment	2020			2021		
		Yield	TSS	A	Yield	TSS	A
Amiral	T1, control	10235 i	12.92 c	1.24 b	11125 i	13.23 c	1.42 b
	T2, fertilizer F	10806 h	13.40 bc	1.35 b	11375 i	13.14 c	1.52 a
	T3, NanoFert D	11671 g	14.05 b	1.46 b	12086 h	14.27 b	1.38 b
	T4, NanoFert Z	11530 g	13.82 bc	1.41 b	12194 h	13.82 bc	1.58 a
de Valu	T1, control	12321 f	13.30 c	1.50 a	13006 g	13.38 bc	1.62 a
	T2, fertilizer F	12652 ef	14.10 b	1.58 a	13813 fg	13.26 c	1.48 b
	T3, NanoFert D	13615 e	14.40 b	1.64 a	14676 e	14.03 b	1.75 q
	T4, NanoFert Z	13513 e	14.22 b	1.72 a	14485 e	13.52 bc	1.64 a
Mimi	T1, control	15085 e	12.08 d	0.51 d	15750 d	12.68 cd	0.55 d
	T2, fertilizer F	15752 d	12.35 d	0.53 d	16884 c	13.12 c	0.55 d
	T3, NanoFert D	17222 c	13.28 c	0.48 d	18326 b	13.45 bc	0.58 d
	T4, NanoFert Z	17020 c	13.07 c	0.55 d	18301 b	13.17 c	0.57 d
Catherine Sel. 1	T1, control	16125 b	14.46 b	0.62 c	16938 c	15.24 a	0.65 c
	T2, fertilizer F	16803 ab	14.37 b	0.62 c	18250 b	15.45 a	0.69 c
	T3, NanoFert D	18188 a	14.69 ab	0.65 c	19345 a	15.87 a	0.72 c
	T4, NanoFert Z	17831 a	15.18 a	0.69 c	19126 a	15.72 a	0.73 c

Values with the same letter within a series are not significantly different at *p*  ≤  5%.

**Table 7:** Average monthly temperatures and precipitation from March to August at Research Station for Fruit Growing, Constanța, in 2020 and 2021 and in comparison with the multi-annual average (1975–2015).

Parameter, Year	March	April	May	June	July	August
Temperature, 2020	8.1	10.3	16	21.2	23.8	23.6
Temperature, 2021	4.7	9.2	16.2	20	24.2	23.5
Temperature, multi-annual average	5.8	10.7	16/4	20.8	23.7	22.8
Precipitation, 2020	19	7.2	19.2	39.8	9.6	2.2
Precipitation, 2021	65.8	66.8	87.8	124.5	39.4	17.6
Precipitation, multi-annual average	34.1	34.1	43.7	42.7	69.3	43.6

## Data Availability

Not applicable.

## Supplementary Materials

The following are available online at https://www.mdpi.com/article/10.3390/plants10112395/s1, Figure S1: Location of the fruit trees, apricot and peach, in the orchard of RSFG Constanța. Table S1: Plant protection treatments applied on apricot fruit trees, Table S2: Plant protection treatment applied on peach fruit trees.
