# Supplementary material for: Effects of Siliceous Natural Nanomaterials Applied in Combination with Foliar Fertilizers on Physiology, Yield and Fruit Quality of the Apricot and Peach Trees
Source: Plants (Basel). 2021 Nov 6;10(11):2395. doi: 10.3390/plants10112395 (PMC8618693; doi:10.3390/plants10112395)
Supplement: Supplementary file 1 [file plants-10-02395-s001.zip › plants-1438466-supplementary.pdf]

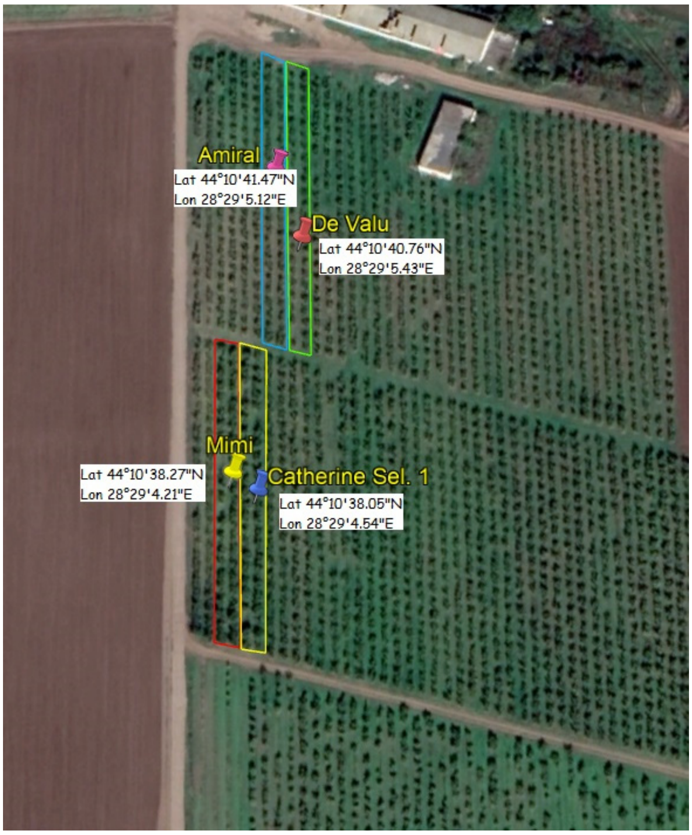

Figure S1. Orchard of Research Station for Fruit Growing (RSFG) Constanța, with the location of the apricot (*P. armeniaca*) cultivars, Amiral and de Valu, and peach (*P. persica*) cultivar, Mimi and Catherine sel 1.

Table S1. The plant protection treatments for apricot cultivars.

Treatment number	Phenological stage (BCCH)	Controlled plant pathogen of pest	Applied product (spraying concentration%)
T1	00 - Dormancy	*Quadraspidiotus perniciosus*, overwintering / resistance forms of plant pathogens and/or pests	Bordeaux mixture 1% + Horticultural oil 0.6%
T2	09 - Green leaf tips visible	*Monilinia laxa, Monilinia fructigena, Alternaria tenuissima. Citospora cincta, Stereum purpureum, Schizophillum comune*	Copper oxychloride 50%, concentration 0.3 %
T3	62 -About 20% of flowers open	*Monilinia laxa, Monilinia fructigena, Stereum purpureum, Stigmina carpophilla.*	Cyprodinil 750g/kg, conc. 0.02%
T4	65 - Full flowering	*Monilinia laxa, Monilinia fructigena, Stereum purpureum, Stigmina carpophilla, Laspeyresia molesta, Anarsia lineatella.*	Difenoconazole 250 g/L, conc. 0.015%
T5	69 - End of flowering	*Monilinia laxa, Monilinia fructigena, Stereum purpureum, Stigmina carpophilla, Laspeyresia molesta, Anarsia lineatella.*	Difenoconazole 250 g/L, conc. 0.015% + Lambda Cyhalothrin 50 g/L, conc. 0.02%
T6	71 - Ovary growing	*Stigmina carpophilla, Podosphaera tridactyla, Limontria dispar, Laspeyresia molesta, Anarsia lineatella, Myzodes persicae*, etc.	Difenoconazole 250 g/L, conc. 0.015% + alpha-cypermethrin and bifenthrin 100 g/L conc. 0.02%
T7	73 - Second fruit fall	*Transhelia pruni spinosae* + defoliating insects	Thiophanate-methyl 700g/kg conc. 0. 07%+

		Stigmina carpophilla, Gnomonia erythrostoma, Podosphaera tridactyla, Alternaria tenuisima.	Acetamiprid 200g/kg conc. 0.02%
T8	75 - Fruit about half final size	*Coryneum beijerinckii, Stereum purpureum, Cytospora cincta, Myzodes persicae* + cicadas insects	Mancozeb 800 g/Kg conc. 0.2 % + Cypermethrin 250 g/L conc. 0.02%
T9	76 - Fruit about 60% of final size	*Coryneum beijerinckii, Stereum purpureum, Cytospora cincta, Myzodes persicae* + cicade.	Cyprodinil 750g/kg, conc. 0.015% + Alpha-Cypermethrin 100 g/L conc. 0.02%
T10	81 - Beginning of fruit coloring	*Monilinia laxa, Monilinia fructigena, Quadraspidiotus perniciosus Anarsia lineatella, Laspeyresia molesta, Carpocapsa pomonella, Tranzschelia discolor.*	Cyprodinil 750g/kg, conc. conc. 0.045% + Cypermethrin 250 g/L conc. 0.02%
T11	Immediately after 89 - Fruit ripe for consumption	*Monilinia laxa, Monilinia fructigena, Quadraspidiotus perniciosus Anarsia lineatella, Laspeyresia molesta, Carpocapsa pomonella*	Thiophanate-methyl 700g/kg conc. 0. 07% + Acetamiprid 200g/kg, conc. 0.02%

Table S2. The plant protection treatments for peach cultivars

Treatment number	Phenological stage (BCCH)	Controlled plant pathogen of pest	Applied product (spraying concentration%)
T1	00 - Dormancy	*Quadraspidiotus perniciosus*, overwintering / resistance forms of plant pathogens and/or pests	Bordeaux mixture 1% + Horticultural oil 0.6%
T2	01- Beginning of bud swelling	*Taphrina deformans, Cytospora cincta, Sphaerotheca pannosa, Stereum purpureum, Stigmina carpophilla.*	Copper oxychloride 50%, concentration 0.3 %
T3	09 - Green leaf tips visible	*Taphrina deformans, Cytospora cincta, Sphaerotheca pannosa, Stereum purpureum*	Bordeaux mixture, conc. 0.5 %
T4	67 - Flowers fading	*Taphrina deformans, Cytospora cincta, Sphaerotheca pannosa, Stereum purpureum, Stigmina carpophilla., Myzus persicae, Anarsia lineatella. Tortrix viridana, Archips podana, Grapholita molesta.*	Difenoconazole 250 g/L, conc. 0.015% + Lambda Cyhalothrin 50 g/L, conc. 0.02%
T5	69 -End of flowering	*Taphrina deformans, Sphaerotheca pannosa, Stigmina carpophilla, Anarsia lineatella, Grapholita molesta*	Difenoconazole 250 g/L, 0.02% + Zeta Cypermethrin 100 g/L conc. 0.01%
T6	73- Second fruit fall	*Cytospora cincta, Sphaerotheca pannosa, Stigmina carpophilla, Tortrix viridana, Archips podana, Myzodes persicae.*	Thiophanate-methyl 700g/kg conc. 0. 07% + Acetamiprid 200g/kg, conc. 0.02%
T7 și T8	75 - Fruit about half final size	*Forficula auricularie, Myzodes persicae, Anarsia lineatella, Stigmina carpophilla, Sphaerotheca pannosa*	Thiophanate-methyl 700g/kg, conc. 0.07 % + Cypermethrin 250 g/L, conc. 0.02%

T9	78- Fruit about 80% of final size	*Monilinia sp,*<br>*Stigmina carpophila, Xanthomonas campestri pv.pruni, Sphaerotheca pannosa var. persicae*	Cyprodinil 750g/kg, conc. 0.05%
T10	Immediately after 89 - Fruit ripe for consumption	*Taphrina deformans ,*<br>*Sphaerotheca pannosa var. Persicae*<br>*Stigmina carpophila, Hyphantria cunea G2, Quadraspidiotus perniciosus, Myzus persicae,* aphides, defoliating insects, etc.	Thiophanate-methyl 700g/kg conc. 0. 07% +<br>Acetamiprid 200g/kg, conc. 0.02%
T11	97 - All leaves fallen	*Taphrina deformans ,*<br>*Sphaerotheca pannosa var. Persicae*<br>*Stigmina carpophila, Hyphantria cunea G2, Quadraspidiotus perniciosus,Myzus persicae,* aphides, defoliating insects, etc.	Copper oxychloride 50%, concentration 0.3 %
